# A pragmatic approach to infants with Robin sequence: a retrospective cohort study and presence of a treatment algorithm

**DOI:** 10.1007/s00784-015-1407-6

**Published:** 2015-02-15

**Authors:** Emma C. Paes, Daan P. F. van Nunen, Lucienne Speleman, Marvick S. M. Muradin, Bram Smarius, Moshe Kon, Aebele B. Mink van der Molen, Titia L. E. M. Niers, Esther S. Veldhoen, Corstiaan C. Breugem

**Affiliations:** 1 Department of Plastic, Reconstructive and Hand Surgery, Wilhelmina Children’s Hospital, Utrecht, The Netherlands; 2 Department of Otorhinolaryngology, Wilhelmina Children’s Hospital, Utrecht, The Netherlands; 3 Department of Oral and Cranio-Maxillofacial Surgery, Wilhelmina Children’s Hospital, Utrecht, The Netherlands; 4 Department of Pediatrics, Wilhelmina Children’s Hospital, Utrecht, The Netherlands; 5 Department of Pediatric Plastic Surgery, Wilhelmina Children’s Hospital Utrecht, PO Box 85500, 3508 GA Utrecht, The Netherlands

**Keywords:** Pierre Robin, Mandibular distraction, Tracheotomy, Tongue–lip adhesion, Approach, Treatment, Multidisciplinary team

## Abstract

**Objectives:**

Initial approaches to and treatments of infants with Robin sequence (RS) is diverse and inconsistent. The care of these sometimes critically ill infants involves many different medical specialties, which can make the decision process complex and difficult. To optimize the care of infants with RS, we present our institution’s approach and a review of the current literature.

**Material and methods:**

A retrospective cohort study was conducted among 75 infants diagnosed with RS and managed at our institution in the 1996–2012 period. Additionally, the conducted treatment regimen in this paper was discussed with recent literature describing the approach of infants with RS.

**Results:**

Forty-four infants (59 %) were found to have been treated conservatively. A significant larger proportion of nonisolated RS infants than isolated RS infants needed surgical intervention (53 vs. 25 %, *p* = .014). A mandibular distraction was conducted in 24 % (*n* = 18) of cases, a tracheotomy in 9 % (*n* = 7), and a tongue–lip adhesion in 8 % (*n* = 6). Seventy-seven percent of all infants had received temporary nasogastric tube feeding. The literature review of 31 studies showed that initial examinations and the indications to perform a surgical intervention varied and were often not clearly described.

**Conclusions:**

RS is a heterogenic group with a wide spectrum of associated anomalies. As a result, the decisional process is challenging, and a multidisciplinary approach to treatment is desirable. Current treatment options in literature vary, and a more uniform approach is recommended.

**Clinical Relevance:**

We provide a comprehensive and pragmatic approach to the analysis and treatment of infants with RS, which could serve as useful guidance in other clinics.

## Introduction

Mandibular micrognathia, glossoptosis with subsequent airway obstruction, is the original triad of symptoms described by Pierre Robin in 1923 [1]. By 1934, the frequent association of a cleft palate was noted by him [2]. These features combined are currently known as Robin sequence (RS). RS may be an isolated condition, but an associated syndrome is present in about 45–80 % of cases [3]. Reported incidences are estimated to be 1:8000 to 1: 14000 births [4–6]. Symptoms of the condition include varying degrees of upper airway obstruction (UAO) and feeding problems, leading to failure to thrive [7, 8]. Mortality rates vary from 0 to 26 % and are most usually caused by severe UAO leading to obstructive apnea and secondary cardiac problems [8].

Infants born with RS have been treated with numerous different methods [9]. Most airway management strategies initiate treatment with positional change [7]. With an inadequate response, nonsurgical interventions, such as the use of a nasopharyngeal airway [10, 11] or a palatal plate [12–15], are commonly pursued. Still, in some cases, there can be more severe respiratory obstruction or failure to thrive, necessitating some other form of intervention [16]. This decision-making process can be challenging for caregivers. To date, many authors have described their preferred surgical techniques, such as tongue–lip adhesion (TLA) [17, 18], tracheotomy [19, 20], subperiosteal release of the floor of the mouth [21, 22], or mandibular distraction osteogenesis (MDO) [23, 24].

Currently, guidelines are lacking, and there is a paucity and discrepancy of information in the medical literature on how specific decisions are made. The rationale for the choice of a specific approach is often not or only scantily addressed. It is known that physicians often utilize a treatment method that was learned during their residency period and often continue with this approach [25]. Furthermore, the surgeon’s preference varies between different specialties [25]. Especially in the treatment regimen of this heterogenic disorder, were a multidisciplinary approach is inevitable, all of this may lead to unnecessary interventions and a potential delay in definitive treatment [7, 26].

The objective of this study is to present a treatment algorithm based on our experience of airway management in infants with RS. The rationale of specific decisions will be covered. This will provide a comprehensive guidance for a designated treatment strategy and contributes in optimizing the care of infants with RS.

## Material and methods

All infants <1 year old diagnosed with RS, who have been treated at the Wilhelmina Children’s Hospital Utrecht, The Netherlands, over 16 years (1996–2012), were included in this retrospective cohort study. Ethics committee approval was obtained. RS was defined as signs of airway obstruction and presence of micrognathia. Information about duration of admission and treatment outcome with a follow-up of at least 1 year was extracted from medical records. Moreover, demographic characteristics, performed diagnostics, interventions, and treatment approach were critically analyzed. A subdivision between the nonisolated RS infants (i.e., diagnosis of an additional syndrome related to RS or of other associated anomalies or chromosomal defects not directly related to the features of RS) and isolated RS infants (i.e., only the features of RS without any additional anomaly) was made. Independent samples *t* test and Mann–Whitney *U* test were performed (IBM SPSS Statistics 20.0, IBM Inc., New York, USA).

Subsequently, a literature search to find existing algorithms covering the approach to infants with RS was performed. The search was performed in January 2014 without time limits. Similar keywords were used in the Embase, Medline, CINAHL, Cochrane Library, and Google Scholar databases [“(pierre) robin syndrome/sequence” and “algorithm(s),” “approach,” “(airway) management,” “intervention,” “regimen,” or “treatment”). Only articles that included a clear description of the patient group, performed examinations, decisional factors, and performed interventions were included. Moreover, concise, state-of-the-art reviews suggesting a treatment approach were included. The bibliographies of the selected studies were hand-searched for any additional articles. The search and inclusion process was performed by two authors (E.P. and B.S.).

Finally, the pragmatic approach from our institution is presented in a schematic way.

## Results

### Retrospective cohort analyses

From 1996 to 2012, 75 infants diagnosed with RS were treated in our institution. Baseline characteristics are summarized in Table 1. Mean follow-up was 7.4 years (range, 1–17). Fifty-two percent (*n* = 39) were female. Seventy-two patients (97 %) had a cleft palate. The minority of cases (43 %, *n* = 32) had an isolated form of RS. In one third of the cohort (31 %, *n* = 23), an associated syndrome was present, Stickler (*n* = 11, 48 %) being the most common. In a quarter (26 %, *n* = 20), additional anomalies or chromosomal defects were identified, which were not directly related to a syndrome associated with the features of RS.

**Table 1:** Baseline characteristics of infants with RS patients treated in the Wilhelmina Children’s Hospital 1996–2012

Patients	Number of patients (%)	Female	Male	Median age of presentation in days (IQR)	Gestational age in days	Mean birth weight in grams (SD)	Presence of CP (%)	CP type^b^ (%)
Isolated RS	32 (43)	20	12	10.0 (5–17.75)	275(median), 270(p25), 282 (p75)	3135 (789)	97	I (0); II (16); III (58); IV (26)
Nonisolated RS	43 (57)	19	24	8.0 (1.25–32.75)	277(median), 273 (p25), 282 (p75)	3237 (553)	98	I (3); II (24); III (56); IV (17)
Syndromic RS	23	10	13	7.5 (1–17.75)	279 (median), 273 (p25), 281 (p75)	3314 (512)	100	I (4); II (13); III (61); IV (22)
Stickler syndrome	11							
Treacher Collins syndrome	2							
Spondyloepiphyseal dysplasia	2							
4q deletion syndrome	1							
Van der Woude syndrome	1							
Osteopathia striata with cranial sclerosis	1							
EEC syndrome^a^	1							
Goldberg–Shprintzen syndrome	1							
Yunis–Varon syndrome	1							
Auriculo–Condylar syndrome	1							
Hemifacial microsomia	1							
RS with other associated anomalies or chromosomal defects	20	9	11	10.5 (2–62.75)	275 (median), 272 (p25), 282 (p75)	3149 (597)	95	I (0); II (37); III (53); IV (10)

*RS* Robin sequence, *SD* standard deviation, *CP* cleft palate, *IQR* interquartile range

^a^EEC syndrome, ectrodactyly–ectodermal dysplasia–cleft syndrome

^b^CP type: I, submucous cleft or bifid uvula; II, soft palate; III, soft palate and segment of the hard palate; IV, soft palate and hard palate up to incisive foramen

**Fig. 1:**
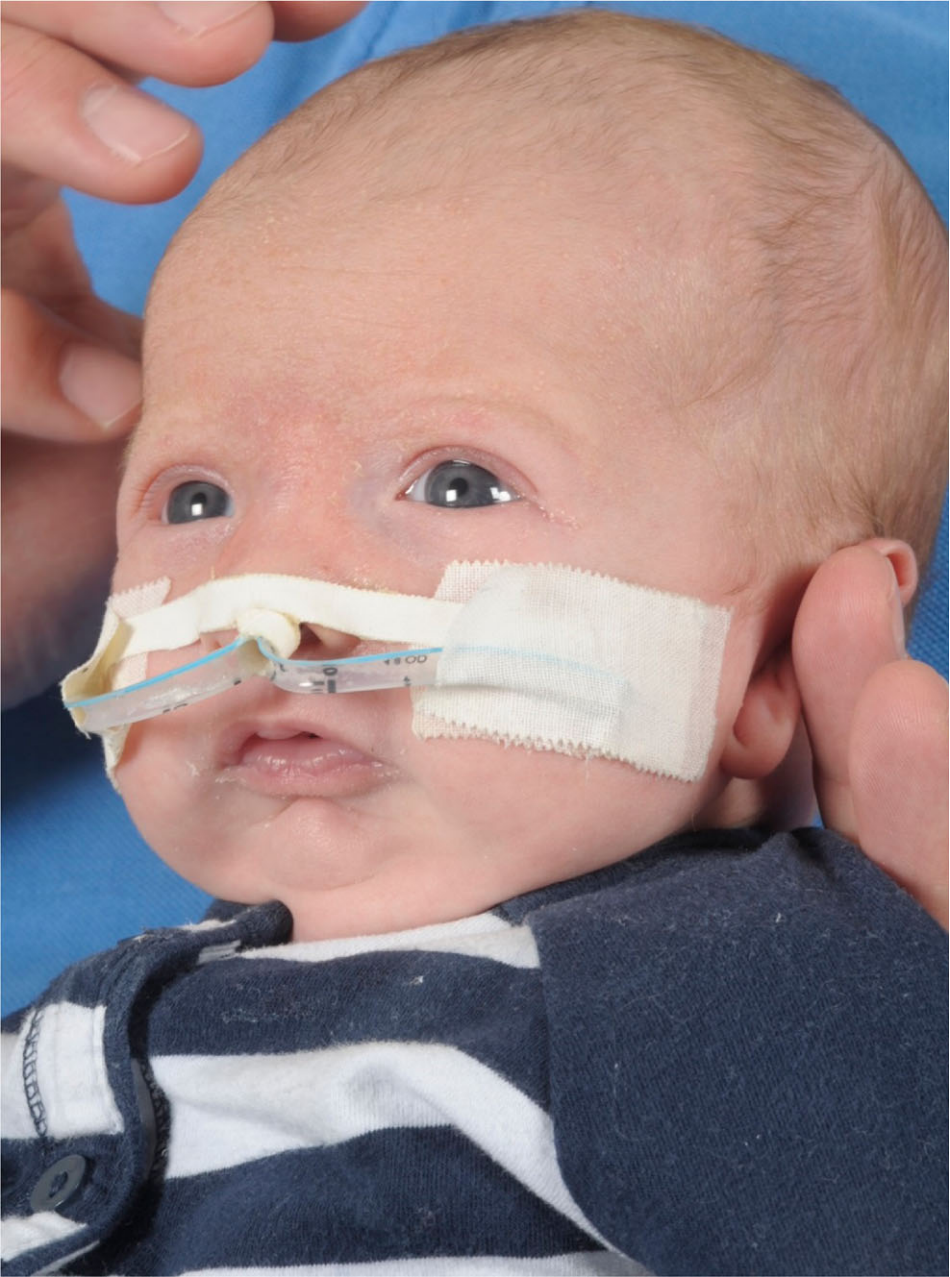
Use of a nasopharyngeal airway as conservative treatment option in a 1-month-old infant with RS

The majority (59 %, *n* = 44) of the infants admitted to our hospital could be successfully managed conservatively (Table 2). This consisted of side/prone positioning, temporary supplemental oxygen or usage of continuous positive airway pressure (CPAP), a mayotube or nasopharyngeal airway (NPA) (Fig. 1). In 41 % (*n* = 31), a surgical intervention was pursued, at a mean age of 50 days (SD, 55). Until 2006, this consisted of TLA whenever possible. If TLA failed, or there was a (sub)glottic pathology, a tracheotomy was performed. Since 2006, the primary surgical intervention for UAO caused by a supraglottic obstruction is MDO. During the study period, in more than half of the surgically treated cases (58 %, *n* = 18), MDO was pursued; in 19 % (*n* = 6) TLA and in 23 % (*n* = 7), a tracheotomy. Average duration until decannulation after a tracheotomy was 13.4 months (range, 4.1–36.5). More than half of the nonisolated RS infants, compared to only a quarter of the isolated RS, infants needed surgical intervention (53 vs. 25 %, *p* = .014) (Table 2). Moreover, mean duration of admission was significantly shorter in the isolated group than in the nonisolated group (33 vs. 58 days, *p* = .018). Two infants with syndromic RS received two interventions: One patient had a tracheotomy prior to MDO; another needed a tracheotomy directly after release of the TLA. Both were successfully decannulated afterwards.

**Table 2:** Approach to infants with RS treated in the Wilhelmina Children’s Hospital 1996–2012

	Total study group	Isolated RS	Nonisolated RS	*p* value*
Number of patients	75	32 (43 %)	43 (57 %)	
Conservative treatment^a^	44 (59 %)	24 (75 %)	20 (47 %)	0.014
Surgical treatment^b^	31 (41 %)	8 (25 %)	23 (53 %)	0.014
MDO	18	6	12	
TLA	6	1	5	
Tracheotomy	7	1	6	
Mean age at surgical intervention in days (SD)	50 (55)	57 (42)	47 (60)	0.620
Mean duration of admission in days (SD)^c^	48 (43)	33 (35)	58 (45)	0.018
Conservatively treated group (SD)	30 (30)	24 (32)	35 (27)	0.285
Surgically treated group (SD)	73 (46)	55 (35)	80 (48)	0.163
Nasogastric tube	58	20 (63 %)	38 (88 %)	0.009

*MDO* mandibular distraction osteogenesis, *TLA* tongue lip adhesion, *SD* standard deviation

*p < 0.05 was considered statistically significant

^a^Side or prone positioning, supplemental oxygen, mayotube, or nasopharyngeal airway

^b^The first surgical intervention was counted

^c^Total duration of all hospital admissions related to airway or feeding problems in the first year of age

Seventy-seven percent of all infants received temporary nasogastric (NG)-tube feeding during an average of 247 days. Average duration of NG-tube feeding was significantly longer in the surgically treated group than in the conservative treated group (resp. median: 72 days; mean rank: 30.5 vs. median: 21 days mean rank: 19.7, *p* = .008). Presence of NG-tube feeding showed a highly significant relation with mean duration of admission (*p* = .000); patients who had received NG-tube feeding were longer admitted in the hospital (44.2 days; SD, 39.1) as compared to patients who had not received NG-tube feeding (4.1 days; SD, 3.6).

Fourteen infants needed temporarily endotracheal intubation prior to surgical intervention, due to severe respiratory distress. Eleven were successfully extubated after surgery. Six infants (8 %), all syndromic RS, ultimately died due to cardiac or pulmonary pathology at a mean age of 416 days (44 days–3 years). All had been immediately intubated within several days after birth. One child was diagnosed with 4q deletion syndrome and received a TLA 23 days after birth and, subsequently, a tracheostomy 52 days after birth. He died due to a cardiac arrhythmia 10 months after the surgery. An infant with Yunis–Varon syndrome underwent TLA after 20 days but could not be extubated, and ultimately died after 41 days due to severe respiratory obstruction. Another infant with Treacher Collins who was primarily treated successfully with MDO 2 weeks after birth, died at almost 2 years of age due to aspiration pneumonia. A child with spondyloepiphyseal dysplasia died 44 days after a tracheotomy due to cardiac failure. Another child with psychomotor retardation, recurrent feeding difficulties, and an atrial and ventricle septum defect died possibly due to a cardiac problem at the age of 9 months. At the request of the parents, no autopsy was performed. Finally, a patient with severe psychomotor retardation, blindness, epilepsia, gastroesophageal reflux, and recurrent pneumonias died at 3 years of age due to sepsis en respiratory insufficiency after an aspiration pneumonia.

### Literature review

The literature search yielded a total of 393 articles. Duplicates were excluded, and abstracts were further analyzed for relevance. Five literature studies [7, 9, 16, 27, 28], 25 retrospective case studies [8, 10, 14, 19, 20, 29–48] and one prospective cohort study [49] fulfilled our selection criteria and were included for further analysis. There was final agreement between the two authors regarding the inclusion process. A summary of the approach described in these articles is listed in Table 3.

**Table 3:** Approaches described in current literature until January 2014

Study	Population^a^	Performed examination^b^	Indication for (surgical) intervention	Type of intervention^c^ (%)	
Abel et al. (2012) [10]	*N*: 104 MG, Gl, CP iRS, sRS	Overnight sleep study Microlaryngobronchoscopy when Tr was considered	Moderate ( ≥3 clusters, ≥3 sPO_2_, 80–85 %) or severe ORD (≥3clusters, ≥3 sPO_2_, <80 %) not responding on positioning and NPA	Tr	19 %
Augarten et al. (1990) [43]	*N*: 8 MG, Gl, CP	Monitoring of vital parameters, blood gases and weight gains Lateral neck radiographs	Respiratory rates ≥60/min, requirement of ≥60 % O_2_, PaO_2_ ≤ 65 mmHg and PaCO_2_ ≥ 60 mmHg or acidemia, despite positioning	TLA Tr if no improvement after TLA	38 %
Benjamin et al. (1991) [19]	*N*: 26 MG/RG, Gl iRS, sRS	Pulse oximetry Laryngoscopy before endotracheal intubation	Oxygen saturation <90 % for >10 % of the time not improving by position or NPA	Endotracheal intubation Tr if this fails, to bypass obstruction	23 %
Bull et al. (1990) [30]	*N*: 21 RS (not specified) iRS, sRS	Modified PSG during 2 h When indicated: nasoendoscopy, airway fluoroscopy, upper GI radiographs and scintiscan and head CT Suggestive/gastroesophageal reflux but normal radiographic studies: pH probe during PSG	↑ End tidal CO_2_ or uncorrectable desaturation (<90 % in >5 % of the sleep time or <80 % in 1 % of the sleep time) with 2 L nasal O_2_ Continued failure to thrive despite nutritional and oxygen supplementation	TLA or Tr	48 %
de Buys Roessingh et al. (2007) [29]	*N*: 48 MG/RG, Gl, ORD, CP iRS, sRS	Pulse oximetry Serial blood gas (every 2 days) Nasoendoscopy, bronchoscopy, pH probe PSG if monitoring shows bad results	Desaturation < 90 % with clinical evidence of respiratory distress or chronic CO_2_ retention (BE > 6.5) despite CPAP followed by NPA and palatal plate	TLA, Tr	0 %
Caoutte Laberge et al. (1994) [31]	*N*: 125 MG/RG, Gl, ORD iRS, sRS	Serial blood gas measurement Oxygen saturation monitoring Modified PSG (according to Freed et al. 1988)	PO_2_ < 60 mmHg or PCO_2_ > 50 mmHg	Subperiosteal release of the floor of the mouth musculature or TLA; Tr if no relieve of UAO	18 %
Cheng et al. (2010) [32]	*N*: 20 MG, Gl, ORD	Continuous oxygen saturation measurement Laryngoscopy and bronchoscopy before MDO Preoperative PSG	Extensive periods of desaturations <90 % not responding on CPAP	MDO + TLA	30 %
Cole et al. (2008) [33]	*N*: 39 MG, Gl, CP	Weight gain and saturation monitoring	Moderate to severe respiratory distress when nursed side to side or with NPA	No surgical intervention performed	0 %
Cruz et al. (1999) [34]	*N*: 47 MG, Gl, CP iRS, sRS	PSG, nasoendoscopy Laryngoscopy, and consideration of flexible and or rigid bronchoscopy before invasive treatment Speech/swallow team evaluation using oropharyngeal motility studies	No resolve of the “airway difficulty” with positioning or short-term use of an NPA	TLA Tr in (sub)glottic pathology or other swallowing or neuromuscular difficulties	43 %
Dauria et al. (2008) [35]	*N*: 9 MG, ORD iRS	Laryngoscopy and bronchoscopy 3D CT before distraction	Failure of positioning or NPA	Tr MDO if no compounding pathology and /or gestational age >39 weeks	44 %
Evans et al. (2011) [7]	Literature study	Modified PSG is important in early infancy for CO_2_ retention in addition to hypoxemia or desaturation, overnight full PSG may have a role when clinical picture is not clear Laryngoscopy and bronchoscopy	No airway stability (abnormal oxygen saturations, carbon dioxide levels, presence of work of breathing and signs of airway obstruction) maintained by positioning or NPA	Temporarily endotracheal intubation TLA/MDO: Single level tongue base obstruction Tr: >1 level of obstruction or not a candidate for TLA/MDO	–
van den Elzen et al. (2001) [8]	N: 74 MG, CP, Gl iRS, sRS	Continuous pulseoximetry PSG on indication, not routinely performed	Hypoxia (continuous and persistent SpO_2_ levels <90 %) not responding on positioning or NPA	Endotracheal intubation Tr (if no successful extubation within 4–6 weeks or after repeated intubations)	15 %
Freed et al. (1988) [36]	*N*: 6 MG, CP, Gl, ORD iRS, sRS	Transcutaneous oxygen and transcutaneous carbon dioxide levels during a minimum of 8 h (range 8–18 h) Modified PSG Studied in lateral, prone and supine position for ≥45 min	Average oxygen levels <60 mmHg and CO_2_ levels >60 mmHg during ≥8 h Any O_2_ level <80 % Obstructive episodes on PSG	TLA	67 %
Gangopadhyay et al. (2012) [44]	Not mentioned	Continuous pulse oximetry PSG can be a useful tool	Inadequate results on sleep studies and poor weight gain despite positioning, supplemental O_2_ and NPA	TLA or MDO (both options discussed with parents and team)	Not mentioned
Gilhooly et al. (1992) [37]	*N*: 15 MG, Gl, ORD iRS, sRS	4-channel PSG including ECG	“Event of obstruction” of ≥15 s during sleep or quiet activity or shorter episodes associated with ↓ HR < 80 BPM or sPO_2_ < 85 % despite positioning	TLA	40 %
Glynn et al. (2011) [20]	*N*: 69 MG, Gl, CP iRS, sRS	Nasoendoscopy Continuous oxygen saturation monitoring for 24–36 h Hearing assessment with otoscopy, tympanometry, visual response and pure audiometry Microlaryngobronchoscopy before Tr	SpO_2_ < 90 % >5 % of the time, despite positioning and NPA	Endotracheal intubation Tr if attempts to extubate fail	14 %
Hoffman et al. (2003) [46]	*N*: 72 MG, Gl, ORD, CP iRS, sRS	Clinical examination PSG Bronchoscopy	Average transcutaneous O_2_ < 60 mmHg/CO_2_ > 50 mmHg, SpO_2_ < 880 %, and/or obstructive episodes on sleep study despite positioning and supplemental oxygen	TLA Tr for (sub)glottic pathology	35 %
Jarrahy et al. (2012) [27]	Literature study	CT scan, manometry, electromyography, 24 h pH monitoring, and nuclear medicine imaging to evaluate presence of reflux Nasoendoscopy pre- and postoperative, “sleep evaluation”	Failure of positioning/NPA orunsuitable airway for a trial of nonsurgical management	Subperiostal floor of mouth release TLA, MDO, Tr	–
Kochel et al. (2010) [14]	*N*: 7 MG, Gl, ORD +/− CP iRS, sRS	Nasoendoscopy Continuous pulse oximetry Blood gas analyses	Clinical signs of respiratory distress (i.e., agitation, dyspnea, tachypnea, intercostal recession, etc.) or oxygen desaturation or respiratory acidosis in blood gas analyses	Orthopedic oral appliance with/without extension (posterior, extra oral or pharyngeal tube)	100 %
Van Lieshout et al. (2013) [38]	*N*: 59 MG/RG, ORD iRS, sRS	PSG (in all infants with ORD despite prone positioning or with persistent feeding difficulties) Nasoendoscopy on indication	Failure of prone positioning and respiratory support (NPA, CPAP, and/or oxygen supplementation)	Tr and/or MDO	7 %
Mackay et al. (2011) [16]	Literature study	Evaluation of desaturation occurring spontaneously, during feeding and sleep Nasoendoscopy, bronchoscopy PSG, pH monitoring, CT scan and cephalometrics	Persistent obstruction despite positioning or NPA	TLA MDO (if TLA fails) Tr (if MDO fails)	–
Marques et al. (2000) [49]	*N*: 62 RG, Gl, ORD iRS, sRS	Nasoendoscopy Continuous pulse oxymetry	SpO_2_ < 90 %, increasing respiratory effort and/or no removal of NG tube possible despite NPA within 15 days	TLA (type 1 obstruction) Tr (type 3 or 4 obstruction, or no improvement after TLA/NPA)	35 %
Poets and Bacher (2011) [9]	Literature study	Clinical observation PSG	Significant UAO during sleep, defined as a mixed-obstructive apnea index (MAOI) > 3 in a sleep study	Pre-epiglottic baton plate	–
Schaefer et al. (2003) [41], Schaefer et al. (2004) [42]	*N*: 21 max–min. discrepancy of >3 mm, Gl, +/− CP	Pulse oximetry for ≥12 h, PSG (continuous monitoring oxygen saturation, end-tidal CO_2_ and EEG during sleep), nasoendoscopy and bronchoscopy before invasive intervention	Any single saturation below the 80 % or PO_2_ < 90 % for >5 % of the monitored time despite positioning	TLA MDO (if TLA fails) Tr if no response to TLA/MDO or (infra)glottic problem present	57 %
Scott et al. (2012) [28]	Literature study	Nasoendoscopy PSG if no life threatening airway compromise is present Serial capillary blood gases (to document a trend of elevated or increasing carbon dioxide levels) Continuous-pulse oximetry and cardiac monitoring	Signs of upper airway obstruction despite prone- or side positioning or NPA	TLA, Tr, MDO	–
Thouvenin et al. (2013) [48]	*N*: 141 RG, Gl, CP iRS, sRS	Continuous monitoring of cardiac and respiratory rhythms, regularly check of transcutaneous PO_2_ and PCO_2_ levels. PSG on indication Karyotype assay, echocardiography, skeletal radiography, ophthalmologic examination	Oxygen saturation < 90 % for >5 % of the time or saturations < 80 % not responding on positional changes or NPA	Tr	Not mentioned
Tomaski et al. (1995) [47]	*N*: 90 MG, Gl, CP iRS, sRS	Flexible fiberoptic nasopharyngolaryngoscopy, cardiac and pulmonary evaluation, chest radiogram, electrocardiogram, ophthalmologic and genetics consultation PSG, continuous pulse oximetry, and apnea monitoring Pre-op: lateral X-ray, rigid direct laryngoscopy and bronchoscopy	Positioning and NPA are not successful in relieving airway obstruction	Tr	12 %
Wagener et al. (2003) [39]	*N*: 22 MG, ORD, Gl, CP iRS, sRS	Continuous oxygen saturation monitoring	Severe UAO (cyanotic attack, transcutaneous oxygenation > 90 %, PCO_2_ < 50 mmHg) not responding on positioning or NPA	No surgical intervention necessary	0 %
Vyas et al. (2008) [40] Kohan et al. (2010) [45]	*N*: 149 MG, ORD iRS, sRS	PSG Radionuclide milk scan (severity of gastroesophogeal reflux and gastric emptying) with 24-h pH probe (in indeterminate results) and laryngobronchoscopy	Intubation at birth necessary, failed extubation or failed conservative treatment (prone positioning or NPA)	MDO Tr if: 1. Central apnea 2. Severe gastroesophageal reflux 3. Other airway lesions	78 %

*MG/RG* micrognathia/retrognathia, *Gl* glossoptosis, *ORD* obstructive respiratory distress, *CP* cleft palate, *iRS* isolated Robin sequence, *sRS* syndromal Robin sequence, *PSG* polysomnography, *Tr* tracheotomy, *TLA* tongue lip adhesion, *MDO* mandibular distraction osteogenesis

### Wilhelmina Children’s Hospital approach

**Fig. 2:**
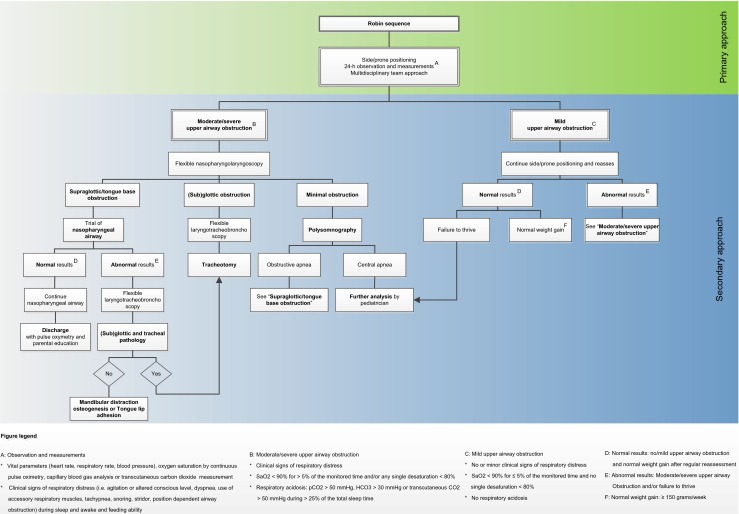
Algorithm of the institutional approach to infants with Robin sequence

Our treatment algorithm is presented in Fig. 2. Infants diagnosed with RS are initially treated in prone or side position when their condition allows it. Prior to any decision making, the patient is observed for at least 24 h. Monitoring of vital parameters, measurements of oxygen saturation by continuous pulse oximetry, capillary blood gas analysis, and more recently transcutaneous carbon dioxide measurements (Tosca®) are performed [50–52]. Observation of clinical signs of respiratory distress during sleep and awake, as well as feeding ability, are documented by experienced nursing and medical staff. We consider oxygen saturations of <90 % for >5 % of the monitored time and/or any single desaturation <80 % as a sign of UAO [39, 41, 42]. Blood gas analysis revealing respiratory acidosis (pCO_2_ > 50 mmHg, HCO_3_ > 30 mmHg) or transcutaneous CO_2_ > 50 mmHg during >25 % of the total sleep time is indicative of hypoventilation [53]. Results are discussed in a multidisciplinary setting consisting of at least a pediatrician, plastic surgeon, otolaryngologist, and a pediatric intensive care specialist after 24–48 h of monitoring. A clinical geneticist is always consulted. Based on the observations and measurements, patients are divided into mild UAO or moderate/severe UAO. These characteristics are described in Fig. 2.

Patients with RS with *mild UAO* remain closely monitored in prone or side position (Fig. 2). Depending on the clinical condition, measurements are repeated and reassessed. Poor weight gain is defined as <150 g/week [41]. In these cases, further analysis by a pediatrician is indicated, and NG-tube feeding may be necessary [37, 54]. If the before-mentioned measurements remain normal, patients will be discharged after the parents are sufficiently instructed. Pulse oximetry is continued at home for an average of 3 months, and at least once a month, the pediatric outpatient department is visited.

**Fig. 3:**
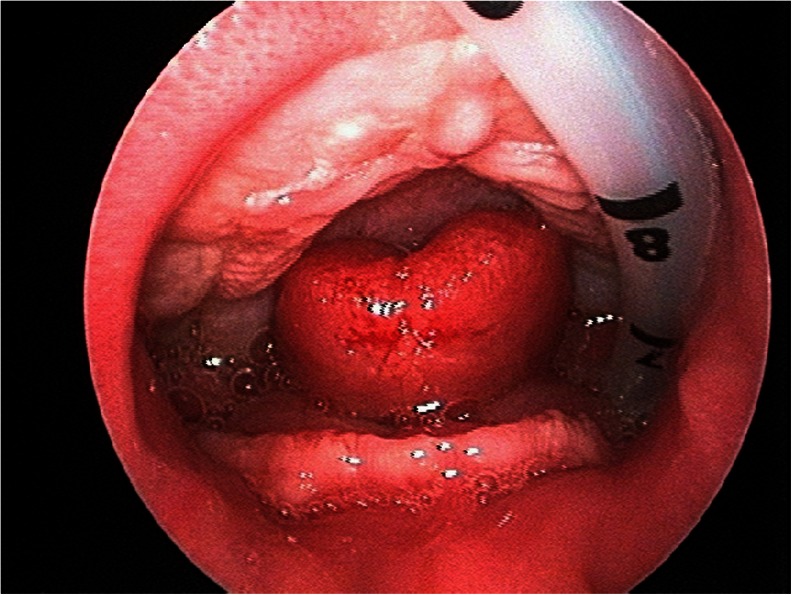
Example of glossoptosis evaluated by direct flexible laryngoscopy

**Fig. 4:**
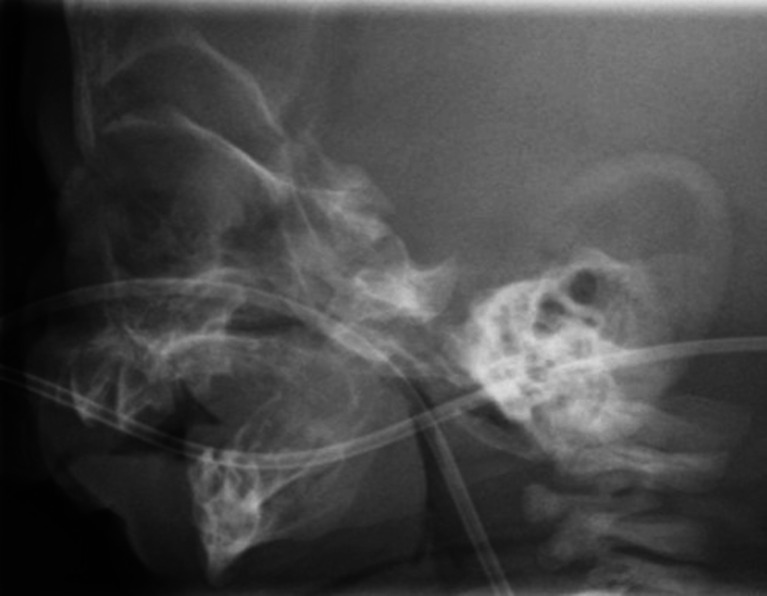
Example of micrognathia seen on lateral X-ray

In *moderate to severe UAO*, the location of the airway obstruction should be investigated by direct flexible laryngoscopy to localize the site of obstruction and to identify possible other airway comorbidities that would influence the decision-making process [55] (Fig. 2). True glossoptosis or other supraglottic obstruction can be diagnosed by this measure (Fig. 3). If the clinical symptoms cannot or only partially be explained by the visible airway obstruction, an overnight polysomnography (PSG) is warranted. Hereby, central apneas, mixed apneas, or episodes of obstructive apnea can be diagnosed, as the glossoptosis tends to be a dynamic problem and could not be identified with laryngoscopy. If substantial central or mixed apnea is detected, a specialist in pulmonary or sleep medicine is consulted. Once the diagnosis of a supraglottic/tongue base obstruction is made, an NPA or mayotube is inserted to maintain a patent airway, and the infant is closely monitored. It is important to mention that other options for conservative treatment, such as orthopedic appliances (like palatal plates or the pre-epiglottic baton plate), have been described to date [12–14]. However, these are not implemented in our algorithm as we are currently not familiar with the use of it in our institution. In the most *severe cases of UAO* (i.e., micrognathia with severe clinical signs of respiratory obstruction, any single desaturation <80 % or severe respiratory acidosis despite positioning) immediate MDO or TLA could be anticipated. However, in our institution, we advocate a trial period of NPA prior to any surgical measure. Depending on the clinical condition of the infant, the case is then reassessed in our team after several days of continuous and cautious monitoring. If earlier-mentioned measurements and observations are normal and the infant shows sufficient weight gain, NPA treatment will be continued. If the infant shows deterioration despite NPA, the surgical options will be discussed with the parents. Until 2006, either TLA or tracheotomy was performed. However, after 2006, MDO has become our surgical procedure of preference when a supraglottic obstruction and a true micrognathia together with a normal functioning temporomandibular joint are present [56]. Before surgery is pursued, other pathology should be ruled out by flexible laryngotracheobronchoscopy. Moreover, radiological assessment of the mandible with a lateral X-ray or CT scan is obtained (Fig. 4). Our performed technique with a resorbable internal distraction apparatus has been described previously [23, 24]. Occasionally, when patients do not have a very small mandible but evident glossoptosis is present, TLA is performed. Still, as it is difficult to accurately assess the mandibular size in infants, often, both procedures and their (dis)advantages are discussed in our team and with the parents. After surgery, vital parameters and blood gasses with pulse oximetry should be regular reassessed.

In our cohort, 29 infants (39 %) suffered *mild* RS according to our classification. Of these, four (14 %) had to be subsequently treated according the *moderate*/*severe* limb of the algorithm, of whom one patient ultimately received a tracheostomy. Forty-six (61 %) of the admitted infants were initially classified as *moderate*/*severe* RS and treated accordingly. Of these, 30 infants (47 %) were treated surgically.

## Discussion

The current study was performed due to the paucity of treatment algorithms for infants with RS in the literature. A plethora of different treatment modalities is suggested, but decisions and rational on which the interventions are based are variable and often ambiguous. A more standardized approach to this challenging clinical entity should be used. An efficient strategy with a multidisciplinary approach might decrease mortality and morbidity, as possible respiratory and feeding problems may be more quickly recognized and treated more efficient [31]. By a thorough retrospective analysis of the treatment regime in our institution and a review of the current literature, we have developed recommendations in the form of an algorithm, which could be applied as a guidance for other centers involved in the care for infants with RS.

The understanding of RS is among others hampered by the numerous different definitions that are used to describe this condition [26, 57–59]. Most authors of the analyzed studies use the criteria described by Pierre Robin in 1934, thus presence of micrognathia, glossoptosis, respiratory distress, and a cleft palate (Table 3) [2]. According to others, in our institution, we define the disorder as presence of micrognathia combined with signs of UAO [38]. Sometimes, we encounter difficulty in determining glossoptosis, possibly due to the fact that intra-oral inspection does not immediately have to reveal its presence and no easy applicable scale of measurement exists. Glossoptosis can be a dynamic problem in which the degree of “ptosis” of the tongue, and subsequent upper airway obstruction, varies, depending on the position and state of the infant (for example asleep, during feeding or tired). Moreover, UAO in patients with micrognathia does not necessarily indicate glossoptosis, since other or additional causes for airway obstruction might be present (e.g., neurologic abnormalities, pharyngeal hypotonia, or choanal atresia) in RS [3, 55]. Therefore, we advice to perform a flexible laryngoscopy in cases with moderate/severe UAO to quickly obtain more information about this matter (Fig. 3). Endoscopic findings in RS have been clearly described and graded [55]. Finally, the presence of a cleft palate is not obligatory for the diagnosis, although it was encountered in 97 % of our patients [9, 44].

A common understanding in the literature is to start every treatment of an RS patient with conservative measures. In our series, more than half of the infants (59 %, *n* = 44) could be treated conservatively, which is comparable to others [30, 32, 34, 35, 37, 49]. Conservative treatment usually starts with prone or side positioning, which will reduce airway obstruction at tongue base level by allowing the mandible and tongue to fall forward. Some do advocate side positioning, since prone positioning might obscure signs of respiratory distress and makes it difficult for the parents to interact with their baby [33]. Supplemental oxygen can be provided when necessary by a nasal cannula. When positioning fails, use of an NPA, mayotube, or CPAP are frequently described secondary measures (Table 3). NPA has obtained a lot of interest and revealed good results (Fig. 1) [10, 29, 33, 39]. According to our approach, the majority of the authors starts using an NPA when positioning fails [8, 10, 16, 19, 20, 27–29, 33–35, 38–40, 44, 45, 47–49]. As we have obtained feasible results, we currently use NPA in every infant with significant UAO before a surgical measure is initiated, and no longer apply a mayotube or CPAP. Yet, the exact place of CPAP still needs to be defined in the treatment of RS. Certain drawbacks of NPA are known. Duration of treatment, obstruction, or luxation of the tube, the burden of care for the parents when the child is discharged with NPA, and persistent feeding problems during the treatment have been described [10, 60]. Finally, also other conservative options, such as the custom-made palatal plate or pre-epiglottic baton plate (PEBP), have been described to date [12, 14, 15]. The promising results of a velar extension in the PEBP have been demonstrated in a randomized clinical trial regarding isolated RS infants [15]. It has also revealed positive effects on feeding issues [61]. The PEBP might completely obviate the necessity of a surgical intervention, by noninvasively moving the base of the tongue forward and subsequently widening the oropharynx. It is speculated that this protrusion of the tongue might also stimulate mandibular growth, although this has not yet been proven [13]. Still, when using these orthopedic appliances, it is necessary to have an experience team, including skilled nurses who can guide and train parents in handling the PEBP [9]. As demonstrated in the literature, the training during residency is of paramount importance regarding what technique will be utilized in the institution [25]. However, the country and the supporting medical system will also influence the decision making in what the most useful conservative treatment option will be.

The percentage of infants that need invasive treatment differs from 0 to 78 % [14, 29, 33, 39, 40, 45] in medical literature (Table 3). These varying percentages are illustrative of the difficulty to accurately define at which exact point the infant fails to respond to conservative treatment and a surgical intervention is anticipated. Many authors tend to use cut-off values derived from specific tests such as PSG or blood measurements to determine candidacy for surgery [10, 19, 20, 29–32, 36, 37, 39, 41, 42, 44, 46, 47] (Table 3). While we also take account of oxygen saturations and CO_2_ levels, we strongly recommend that the multidisciplinary team considers all available results including clinical observation and feeding status, when deciding about escalating care (Fig. 2). Standard usage of PSG in the management of RS remains a point of discussion [7, 28, 29, 37, 62]. In accordance to others and as demonstrated in the flowchart, in our institution, PSG is only performed on indication, to exclude central apneas or to quantify more subtle airway obstruction if the clinical symptoms cannot or only partially be explained by the visible airway obstruction (Fig. 2) [7, 19, 20, 29, 37, 39] In the majority of the studies that used PSG routinely, a so-called “modified PSG” is performed using only certain components of PSG [31, 32, 36, 37, 41, 42, 46]. It is mandatory that important matters such as the exact indication, frequency, and the extensiveness of the conducted tests on PSG are clarified in further studies, and strict recommendations for its use can be made.

Forty-one percent of the infants of our cohort needed a surgical intervention, comparable to findings described by others [29, 30, 34, 35, 37, 43, 46]. In the analyzed literature, surgical interventions consist mainly of TLA [20, 28–31, 34, 36, 37, 41–43, 46, 49] or MDO when TLA failed [16, 41, 42] (Table 3). Some recommended MDO as primary measure [35, 40, 45] or a combination of MDO and TLA [32]. Generally, tracheotomy was considered as final option when there was no improvement after TLA and/or MDO [7, 16, 27, 28, 31, 34, 35, 41–43, 46, 49]. Other indications were the presence of a central neurological impairment or coinciding upper airway [i.e. (sub)glottic obstruction or tracheo- or laryngomalacia], cardiac, pulmonary, or gastroesophageal pathology, contributing to the respiratory distress [7, 27, 28, 30, 34, 35, 40–42, 44, 46]. Still, some authors choose tracheotomy as the primary surgical strategy after conservative treatment has failed [10, 19, 20, 47, 48, 63]. In our series, MDO was the primary choice in more than half of the surgically treated cases (Table 2). Until 2006, TLA was our surgical procedure of preference with subsequent tracheotomy if TLA failed or could not be performed due to (sub)glottic pathology. At this moment, we only perform TLA in the rare cases where patients have an obvious glossoptosis without clear micrognathia. Objectively assessing the size of micrognathia is not easy, and currently, the (dis)advantages of both MDO and TLA are discussed in a multidisciplinary team meeting and with the parents. It is important to emphasize the risk of glossoptosis recurring after TLA release [64]. MDO also proves to be more effective than TLA in resumption of normal oral feeding [65]. In our series, all children could bottle feed within 4 weeks after distraction and NG-tube feeding could be stopped [24]. Additionally, since we use a resorbable distracting system, there is no need for a second intervention, while patients with TLA need secondary surgery to release the adhesion [24]. Finally, there is less scar formation after MDO [66]. However, long-term follow-up studies after MDO in RS patients are still scarce. Furthermore, possible damage to the permanent molars in the osteotomy region and mandibular outgrow after MDO remains a point of investigation and discussion. In order to clarify these matters, analyses are currently undertaken at our institution.

Six infants (8 %) of our cohort, all nonisolated RS patients, died after a mean of 416 days (44 days–3 years). Reported mortality rates in the literature vary from 0 up to 26 % [8]. It is important to realize that RS is a heterogenic disorder with numerous causes and also possible co-morbidities, which can aggravate the already present symptoms [44]. An additional syndrome or malformation makes the treatment regime especially challenging and a multidisciplinary approach indispensable. In 75 % of the infants with isolated RS, conservative measures revealed to be sufficient to maintain an adequate airway. In contrast, of the nonisolated infants, only 47 % could be treated conservatively (Table 2). This important, significant finding (*p* = 0.014) demonstrates the relevance of adequate and early genetic analysis. Less favorable airway outcomes are more common in nonisolated RS, potentially attributed to airway and swallowing problems independent of glossoptosis [28, 31, 47, 67]. In addition, mandibular size and morphology vary significantly in syndromic RS [68]. Although we are aware that an associated syndrome could have important consequences for the long-term mandibular outgrowth, this does not influence our initial treatment approach, as the respiratory distress still needs to be treated and will be alleviated by advancing the mandible. However, MDO or other surgical interventions should only be considered after other or additional etiologies of respiratory compromise (such as tracheo-or laryngeomalacia) are ruled out. Furthermore, it needs to be addressed that conservative measures can obtain good results and should always be performed in first instance, also in syndromic RS infants [14].

It is still not fully elucidated what risk factors exist and which infants have an absolute contraindication to receive surgery. Murage and co-workers reviewed the results of 50 infants who were treated with MDO and concluded that the absence of a CP, presence of gastroesophageal reflux, and need for Nissen fundoplication might be associated with failure of MDO [69]. Prematurity, low birth weight, late operation, preoperative intubation, diagnosis of a syndrome, and cardiac and pulmonary abnormalities did not preclude success in appropriately selected patients [69]. On the contrary, others demonstrated that, besides gastroesophageal reflux, also preoperative intubation, late operation (older than 2 weeks), low birth weight, and diagnosis of a syndrome were significant predictive markers of failure of TLA and necessity of a tracheotomy [67, 70]. The cited studies are retrospective and may contain substantial bias. Prospective studies, systematically collecting data, are needed to understand risk factors for failure and success of interventions and to develop evidenced based clinical guidelines to facilitate treatment planning.

Besides the airway problems, feeding difficulties are also a common finding in infants with RS and should be adequately supported [7, 71]. Seventy-seven percent (*n* = 58) of the infants in our series needed NG-tube feeding during an average of 247 days. This comprised of significantly more infants of the nonisolated group (88 %), compared to 63 % of the isolated RS patients (*p* = .009). Duration of NG-tube feeding was also significantly shorter in the conservative treated group (*p* = .008). However, in all cases after MDO, NG-tube feeding could be successfully stopped within 4 weeks postoperatively, independent of the syndromic or nonsyndromic status. Feeding difficulties in RS can have multiple causes [61], on the one hand attributed to the micrognathia, glossoptosis, or the possible concomitant cleft palate, and also to possible additional upper digestive tract motor dysfunction, leading to esophageal motility disorders or reflux disease. Associated cardiac or other complex abnormalities can also lead to compromised growth [7]. In persistent feeding problems, we advice consulting a pediatrician or pediatric neurologist to rule out other pathology.

One of the strengths of the current study is that it gives a clear insight of the treatment in a relatively large cohort of infants with RS, and a structured and pragmatic algorithmic approach including the rationale of the decisions taken. This could be used as guidance in other institutions. Moreover, it provides a clear overview of approaches described in the literature. Limitations include a possible selection bias as this is a tertiary center. In addition, the retrospective nature of the study and the relatively short follow-up period should be emphasized. Finally, we are fully aware that our approach could differ from the supporting medical system and regional habits of other institutions. Other treatment options, such as orthopedic appliances, have been described in the current literature yielding very good results [9, 13, 14]. Yet, in our institution, we do not have experience in using them. However, this does not preclude their beneficial use in infants with RS in other practices.

It should be addressed that many different treatment options could probably be performed on a patient. Each intervention has known (dis)advantages and the outcome depends on multiple factors. Burden of care, treatment duration, long-term complications, and financial implications should be considered [65, 66, 72, 73]. Furthermore, surgical skills and preferences will influence the approach of the treatment center [25]. It is demonstrated that mortality and morbidity are significantly lower in infants treated by the use of a decisional model [40, 45]. However, the choice of a specific treatment for an infant with RS is a continuous and dynamic process, with multiple factors to be regarded and with many caretakers involved. The algorithm, as presented in the current study, should be used as a guideline and not as a rigid decision tree since every patient is unique. Still, using an algorithm, we hope to prevent possible unnecessary proceedings and a potential delay in treatment by helping involved caretakers in decision making. Prospective studies will give us more insight in the outcome of the different strategies, which unfortunately do not exist to date. Using an algorithm, it might be easier to compare the outcome of the different modalities in the nearby future.

## Conclusion

RS is a heterogeneous disorder with numerous different treatment strategies described to date. A pragmatic approach is presented in this manuscript. The management of RS involves a multidisciplinary team approach to achieve a safe airway and adequate growth. We hope that this manuscript will serve as a guidance for caretakers involved in the care for infants with RS and as an impetus for conducting future (preferably prospective) studies.
